# Supplementary material for: Analysis of PPARγ Signaling Activity in Psoriasis
Source: Int J Mol Sci. 2021 Aug 10;22(16):8603. doi: 10.3390/ijms22168603 (PMC8395241; doi:10.3390/ijms22168603)
Supplement: Supplementary file 1 [file ijms-22-08603-s001.zip › Supplemental materials_Analysis of PPARg signaling activity in psoriasis/Pathway models/Models images and html files/PPARG inhibits phenotypes assotiated with psoriasis/33333.html]

hepatitis


# Disease hepatitis

|  |  |
| --- | --- |
| URN | urn:agi-meshdis:Hepatitis |
| Total Entities | 25 |
| Connectivity | 3696 |
| Name | hepatitis |

---

|  |  |
| --- | --- |
| ChildConcepts | chronic active hepatitis |
|  | chronic hepatitis |
|  | drug-induced hepatitis |
|  | acute hepatitis |
|  | cholestatic hepatitis |
|  | cytolytic hepatitis |
|  | granulomatous hepatitis |
|  | human viral hepatitis |
|  | lupus hepatitis |
|  | murine hepatitis |
|  | neonatal hepatitis |
|  | perihepatitis |
|  | viral hepatitis |
|  | post-transfusion hepatitis |
|  | woodchuck hepatitis |
|  | alcoholic hepatitis |
|  | animal hepatitis |
|  | infectious canine hepatitis |
|  | toxic hepatitis |
|  | autoimmune hepatitis |
|  | giant cell hepatitis |
|  | ischemic hepatitis |
|  | experimental hepatitis |
|  | extrahepatic manifestation |
|  | fulminant viral hepatitis |

---

|  |  |
| --- | --- |
| Pathway | PPARG inhibits phenotypes assotiated with psoriasis |
|  | Neighbors of hepatitis |

---

|  |  |
| --- | --- |
| MedScan ID | 9003101 |

---

|  |  |
| --- | --- |
| Alias | hepatic cellular inflammations |
|  | infection in liver |
|  | hepatitic disorder |
|  | sub-acute hepatitis |
|  | Inflammatory Disorders of Liver |
|  | Hepatitis Unspecified |
|  | hepatitic illnesses |
|  | hepato-inflammations |
|  | Inflammatory Liver Diseases |
|  | hepatic inflammatory diseases |
|  | hepatocellular inflammatory syndromes |
|  | liver inflammatory disease |
|  | hepatic inflammation |
|  | Hepatitis |
|  | liver infection |
|  | liver inflammations |
|  | inflammation in the liver |
|  | liver infections |
|  | hepatocellular inflammations |
|  | infections of the liver |
|  | Inflammatory Liver Disease |
|  | Hepatitides |
|  | hepatocellular inflammatory syndrome |
|  | hepatoinflammations |
|  | liver inflammation |
|  | inflammations in the liver |
|  | hepatocellular inflammatory disease |
|  | subacute hepatitis |
|  | hepatitic disorders |
|  | hepato-inflammation |
|  | hepatoinflammation |
|  | hepatitic disease |
|  | hepatitic illness |
|  | hepatic inflammations |
|  | liver inflammatory diseases |
|  | hepatic inflammatory disease |
|  | Nonspecific Hepatitis |
|  | Inflammatory Disorder of Liver |
|  | hepatocellular inflammatory diseases |
|  | Hepatitis Nonspecific |
|  | Inflammatory Disease of Liver |
|  | inflammatory diseases of the liver |
|  | hepatic cellular inflammation |
|  | hepatitic diseases |
|  | hepatocellular inflammation |

---

|  |  |
| --- | --- |
| MeSH ID | D006505 |
|  | M0010205 |

---

|  |  |
| --- | --- |
| MeSH Heading | Hepatitis |

---
